# Machine Perfusion as a Strategy to Decrease Ischemia-Reperfusion Injury and Lower Cancer Recurrence Following Liver Transplantation

**DOI:** 10.3390/cancers16233959

**Published:** 2024-11-26

**Authors:** Karla Bracho Garcia, Ahmed Hussein, Sangeeta Satish, Chase J. Wehrle, Omer Karakaya, Rebecca Panconesi, Keyue Sun, Chunbao Jiao, Eduardo Fernandes, Antonio Pinna, Koji Hashimoto, Charles Miller, Federico Aucejo, Andrea Schlegel

**Affiliations:** 1 Department of Liver Transplantation, Cleveland Clinic Weston Hospital, Weston, FL 33331, USA; 2 Transplantation Center, Department of Surgery, Digestive Disease Institute, Cleveland Clinic, Cleveland, OH 44195, USA; wehrlec@ccf.org (C.J.W.);; 3 Department of Immunology, Lerner Research Institute, Cleveland Clinic, Cleveland, OH 44195, USA

**Keywords:** liver transplantation, hepatocellular carcinoma, tumor recurrence, disease-free survival, hypothermic oxygenated machine perfusion (HOPE), normothermic machine perfusion, mitochondrial injury, ischemia-reperfusion injury, microenvironment

## Abstract

Liver transplantation (LT) is a key treatment for primary and secondary liver cancers, reducing tumor burden with concurrent improvement of liver function. While significant improvement in survival is noted with LT, cancer recurrence rates remain high. Mitochondrial dysfunction caused by ischemia-reperfusion injury (IRI) is known to drive tumor recurrence by creating a favorable microenvironment rich in pro-inflammatory and angiogenic factors. Therefore, strategies that decrease reperfusion injury and mitochondrial dysfunction may also decrease cancer recurrence following LT. Machine perfusion techniques are increasingly used in routine clinical practice of LT with improved post-transplant outcomes and increased use of marginal grafts. Normothermic (NMP) and hypothermic oxygenated machine perfusion (HOPE) provide oxygen to ischemic tissues, and impact IRI and potential cancer recurrence through different mechanisms. This article discussed the link between IRI-associated inflammation and tumor recurrence after LT. The current literature was screened for the role of machine perfusion as a strategy to mitigate the risk of cancer recurrence. Upfront NMP (“ischemia free organ transplantation”) and end-ischemic HOPE were shown to reduce hepatocellular carcinoma recurrence in retrospective studies. Three prospective randomized controlled trials are ongoing in Europe to provide robust evidence on the impact of HOPE on cancer recurrence in LT.

## Simple Summary

Liver transplantation (LT) is a key treatment for liver cancers by reducing tumor burden and improving liver function. While LT offers significant improvement in survival, cancer recurrence rates remain high. Ischemia-reperfusion injury (IRI) caused by mitochondria dysfunction drives tumor recurrence by creating a favorable pro-inflammatory microenvironment. Therefore, strategies that decrease IRI may also decrease cancer recurrence following LT. Machine perfusion techniques are increasingly used in routine clinical practice of LT with improved post-transplant outcomes. Normothermic (NMP) and hypothermic oxygenated machine perfusion (HOPE) provide oxygen to ischemic tissues, and impact IRI and potential cancer recurrence through different mechanisms. This article discussed the link between IRI-associated inflammation and tumor recurrence after LT and examined the role of machine perfusion as a strategy to mitigate the risk of cancer recurrence. Upfront NMP (“ischemia free organ transplantation”) and end-ischemic HOPE were shown to reduce hepatocellular carcinoma recurrence in retrospective studies.

## 1. Introduction

Liver transplantation (LT) is the most effective treatment modality for patients with hepatocellular carcinoma (HCC), as it provides the best oncologic resection while restoring normal liver function [1]. The Milan criteria were established in 1996 and found that patients who underwent LT for HCC with a single tumor less than 5 cm in size or 2–3 tumors less than 3 cm had improved survival [2]. Since then, the Milan criteria have been used to allocate livers to HCC patients. Other criteria have since expanded on the size restrictions of the Milan criteria and incorporated assessment of tumor biology and markers into the selection criteria, with similar post-transplant survival [3]. In addition, extended criteria donor (ECD) organs are frequently used to decrease waiting times for patients outside the Milan criteria with similar post-transplant outcomes [4]. Of note, liver transplantation has also been performed for intrahepatic cholangiocarcinoma (i-CCA) [5], as well as in patients with unresectable secondary liver tumors, such as neuroendocrine tumor (NETs) and colorectal cancer liver metastasis (CLRM) [6,7,8], with improvement in long-term survival.

While long-term survival has improved with LT, tumor recurrence remains a significant problem in this patient population. Recurrence rates for HCC range from 8% to 20% [9,10,11,12]. The recurrence rates are significantly higher for i-CCA [5] (50%), NETs [6] (50–80%), and CLRM [7,8] (80–100%). Risk factors for recurrence are multifactorial and can be divided into three broad categories: tumor biology, donor characteristics, and recipient factors. Tumor differentiation, vascular invasion, size and number of lesions, and level of tumor markers are all noted to impact recurrence [7,13,14]. Donor risk factors including age, cold ischemia time, and donation after cardiac death (DCD) grafts were associated with higher recurrence rates [15]. Obesity, previous chemotherapy, and post-transplant immunosuppression are among the most impactful recipient risk factors associated with higher recurrence rates [16,17,18]. Such donor and recipient factors can cause mitochondrial dysfunction with elevated ischemic-reperfusion injury (IRI) and impaired liver function and regeneration [19]. Numerous experimental studies have assessed the association between IRI and tumor recurrence, as the IRI-phenomenon promotes a microenvironment for tumor cell invasion, migration, and growth [20,21,22].

Organ perfusion techniques convey many benefits and are a relatively new addition to routine LT. Currently, three main machine perfusion techniques are commonly utilized: normothermic regional perfusion, normothermic machine perfusion, and hypothermic machine perfusion. Normothermic regional perfusion (NRP) is utilized in donation after circulatory death donors to re-establish donor circulation to minimize ischemia and promote viability assessment prior to transplantation. Normothermic (NMP) and hypothermic machine perfusion (HOPE) provide controlled re-oxygenation of the liver graft under normothermic (37 degrees Celsius) or hypothermic conditions (8–12 degrees Celsius). However, particularly HOPE promotes organ reconditioning and viability assessment prior to transplant and impacts IRI-associated inflammation upon transplant [23,24,25]. NMP is the only approved technology in the US at present and has been associated with both improvements in pre- and post-transplant outcomes and costs compared to the conventional static cold storage (SCS) [26,27]. However, European studies have demonstrated additional benefits to HOPE in comparison to NMP based on the temperature of re-oxygenation, which relates to improvements in overall complications, NAS, and possibly even cancer recurrence, which will be the primary focus of this review [28,29]. Head-to-head comparison of NMP, HOPE, and NRP using standardized outcomes reporting remains critical for the overall field of transplantation [30,31]. However, specific to this review, HOPE and “ischemia free liver transplant” (IFLT) have shown a decrease in the risk of HCC recurrence, the mechanisms and outcomes of which will be discussed at length [32,33].

This article reviews the mechanisms underlying IRI and its link to ongoing inflammation and related tumor recurrence together with current evidence on the role of machine perfusion. The impact of other transplant-related treatments is also discussed.

## 2. Mechanism of Hepatic Ischemia-Reperfusion Injury

Liver ischemia-reperfusion consists of two distinct phases [34]: (1) the ischemic phase during which cellular dysfunction and injury occur and (2) the reperfusion phase that exaggerates the initial metabolic insult. The reperfusion phase can be further divided into an early phase, which occurs within 2 h after oxygen reintroduction and a late phase that continues up to 48 h [35,36]. The severity of IRI is closely associated with the metabolic liver profile driven by donor comorbidities, lifestyle, and the duration of hypoxia (i.e., warm and cold ischemia). The end-stage liver disease severity, the medical fitness, and recipient co-morbidities contribute further [16,17,18].

Liver ischemia triggers a shift from aerobic to anaerobic metabolism due to a steady decrease in oxidative phosphorylation until the respiratory chain stops entirely with no new adenosine triphosphate (ATP) production [37]. Existing ATP molecules are lost at the same time. This anaerobic metabolism causes intracellular acidosis, resulting in increased activity of the sodium (Na^+^)/hydrogen (H^+^) exchanger and loss of activity of the ATP-dependent sodium (Na^+^)/potassium (K^+^) channel [38]. Intracellular sodium (Na^+^) concentration rises causing cellular swelling and cell death [39]. Calcium (Ca^2+^) homeostasis is disrupted through increased Na^+^/Ca^2+^ exchange and inhibition of the Ca^2+^-ATPase channel, leading to intracellular calcium overload [40,41]. Elevated cytosolic calcium levels activate enzymes including phospholipases and calpains, promoting cell death [42]. Intermediates of aerobic respiration, including succinate and nicotinamide adenine dinucleotide (NADH), accumulate during ischemia [43].

Upon reintroduction of oxygen at transplantation or during machine perfusion, accumulated succinate overwhelms mitochondrial complex II, resulting in an undirected and retrograde electron flow with subsequent reactive oxygen species (ROS) generation and release from complex I [44]. The mitochondrial and cellular succinate levels are the direct driver of such ROS release, which in turn causes downstream inflammation with protein oxidation, lipid peroxidation, DNA damage, and endothelial dysfunction. ROS molecules directly stimulate the release of damage-associated molecular patterns (DAMPs) and cytokines, resulting in local and systemic inflammation [45,46]. Accumulated citric acid cycle (TCA) metabolites, i.e., succinate, also directly impact the immune system and cancer development/recurrence [47].

The acidosis during ischemia inhibits proteolytic enzymes and function of mitochondrial permeability transition (MPT) pores. However, upon reperfusion, the acidosis corrects with re-establishment of ATP production with activation of proteolytic enzymes and subsequent release of cytochrome C through MPT pores. Cytochrome C further contributes to the ROS-induced apoptosis and necrosis [48,49,50], well-known downstream consequences that are also observed during normothermic machine perfusion (NMP) [51].

Mitochondrial dysfunction within hepatocytes and Kupffer cells (KC) largely drives the acute IRI phase, increasing the risk for primary non function (PNF) after liver transplantation. DAMPs (released from hepatocytes and endothelial cells) also activate KC resulting in further ROS generation and cytokine release, including tumor necrosis factor-alpha (TNF-α), interleukin-1-beta (IL-1β), and interleukin-12 (IL-12) [52,53,54,55,56]. This pro-inflammatory milieu causes vascular inflammation and mitochondrial fragmentation within the hepatic sinusoidal epithelial cells (HSEC) [57]. The resulting damage results in an imbalance between endothelin (ET) and nitric oxide (NO), causing endothelial vasoconstriction and diminished blood flow to hepatocytes [58,59]. Systemic NO and ET release after implantation can trigger a drastic drop in recipient blood pressure, contributing to the phenomena of post-reperfusion syndrome (PRS) [60]. Additionally, the pro-inflammatory sinusoidal environment triggers platelet activation that leads to microthrombi formation with further impairment of the liver microcirculation (i.e., secondary regional hypoxia) [61]. Neutrophils and lymphoid cells are main mediators of the late IRI phase through ROS and protease release [62]. Ongoing inflammation with neutrophil, lymphoid cells, and stellate cell activation can induce chronic inflammation resulting in early liver fibrosis and cirrhosis, biliary strictures, and acute cellular rejection [63,64,65].

## 3. The Link Between Ischemia-Reperfusion Injury and Cancer Recurrence

**Figure 1:**
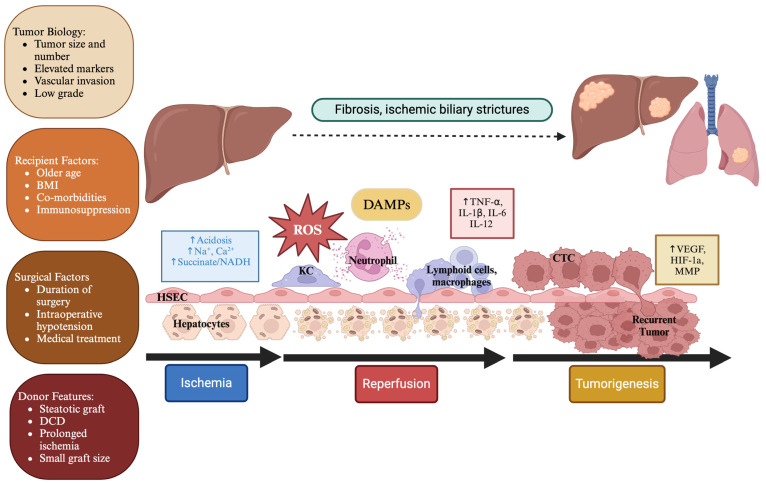
Risk factors and mechanism of ischemia-reperfusion injury (IRI) and cancer recurrence. Tumor biology, recipient factors, surgical factors, and donor features all contribute to recurrence of cancer. Tumor biology such as size, number, vascular invasion, and low-grade differentiation all highlight features of an aggressive tumor with increased risk of recurrence. Recipient factors such as older age, higher body mass index (BMI), underlying co-morbidities, and post-transplant immunosuppression play a role in recurrence risk. Surgical factors such as duration of surgery, intraoperative hypotension, and medical treatment may impact the level of IRI. Finally, donor features, such as macro-steatosis, donation after circulatory death (DCD), prolonged warm and cold ischemia, and small graft size may increase risk of IRI and tumor recurrence. IRI is characterized by two phases: ischemia and reperfusion. During ischemia, cell injury and anaerobic respiration occur resulting in acidosis and buildup of sodium, calcium, and respiration substrates, such as succinate. Upon reperfusion, these factors stimulate release of reactive oxygen species (ROS) at mitochondrial complex I and damage-associated molecular patterns (DAMPs) from hepatocytes, hepatic sinusoidal epithelial cells (HSEC), and Kupffer cells (KC), attracting recipient neutrophils to the site. Inflammatory cytokines such as TNF-α, IL-6, and IL-12 are released stimulating a pro-inflammatory milieu consisting of lymphoid cells and macrophages. Increased vascular endothelial growth factor (VEGF), hypoxia inducible factor (HIF-1a), and matrix metalloproteinases (MMP) allow for remodeling and stimulate angiogenesis. This allows for circulating tumor cells (CTC) to migrate and settle, resulting in recurrence. Significant IRI after liver implantation is also seen in lungs with initial inflammation and edema potentially contributing to the elevated HCC recurrence in lungs after liver transplantation. In addition, this pro-inflammatory environment stimulates early liver fibrosis and ischemic biliary strictures. Created with BioRender.com.

The pro-inflammatory IRI milieu is a favorable environment for tumor cell resettling and cancer recurrence. Both processes share pathways, downstream mediators, and the active role of pro-inflammatory cytokines. With elevated IRI, there is massive release of ROS, DAMPs, and cytokines with associated microvascular dysfunction. This permeates an environment for migration and engraftment of circulating tumor cells (CTC) (Figure 1).

C-X-C motif chemokine ligand 10 (CXCL10) is a major driver of the pro-inflammatory response to IRI [66]. DAMPs activate CXCL10 through Toll-like receptor 4 (TLR4) causing migration of recipient macrophages, monocytes, regulatory B, and regulatory T cells to the liver [67]. In addition, TNF-α, IL-6, and IL-1β release allows for tumor cell implantation and growth by increasing expression of adhesion proteins including E-selectin and upregulation of rho-associated protein kinase-1 (ROCK1), vascular endothelial growth factor (VEGF), and matrix metalloproteinases (MMPs). ROCK overexpression in turn enhances tumor proliferation while VEGF and MMPs promote angiogenesis and extracellular matrix remodeling, thereby creating an environment favorable for tumor engraftment [68,69,70,71].

Tissue hypoxia results in the activation of hypoxia-inducible factor-1a (HIF-1a), a molecule known to promote gene expression for apoptotic and anti-apoptotic factors [71,72]. The overexpression of HIF-1a dysregulates this balance creating an environment selecting cells resistant to hypoxia. HIF-1a alterations have been implicated in cancer cell survival, treatment resistance, and poor prognosis in i-CCA, HCC, and CRLMs [73,74,75]. Other signaling pathways such as PI3K/Akt, NF-κB, and STAT3 also play critical roles in cell survival, proliferation, and immune regulation, further contributing to the favorable microenvironment for cancer cell resettling, proliferation, and recurrence [76,77,78].

## 4. Risk Factors for Elevated Ischemia-Reperfusion Injury and Cancer Recurrence Following Liver Transplantation

Impaired liver metabolism and organ injury begins with the donor status, medical history, and lifestyle. Donor cause of death and medical treatment during admission contribute further as does treatment withdrawal, procurement, and preservation, where livers are exposed to warm and cold ischemia [79]. Warm ischemia time (WIT) is unique to DCD grafts and occurs with variable hypoxia after withdrawal of life support till cross-clamp. This period is particularly deleterious as the liver undergoes hypoxia while still metabolically active, stimulating anaerobic respiration and ROS production. Cold ischemia time (CIT) is present in both DCD and donation after brain death (DBD) grafts. CIT describes the time from cold donor flush to reperfusion in the recipient or on a perfusion device (i.e., end-ischemic machine perfusion). As the temperature drops, the liver’s metabolic drive decreases and less injury results from the ongoing hypoxia. Prolonged WIT (>50 min) and CIT (>10 h) were found to contribute to HCC recurrence, especially in patients with unfavorable tumor biology [80,81]. While DCD grafts experience WIT, the risk of recurrence associated with these grafts is debated and depends on the cell and mitochondrial ability to recover from the ischemic insult. It has been noted that patients with HCC receive a higher proportion of DCD livers versus those without HCC [82]. This is multifactorial with one explanation noting that HCC patients have a lower Model for End-stage Liver Disease score (MELD) and are often medically fitter to better tolerate DCD grafts. Some studies suggest a link between HCC recurrence and DCD grafts [67], with a recent study finding increased recurrence and decreased overall survival (OS) when DCD grafts are used in expanded criteria recipients [3].

Several additional donor factors impact cancer recurrence including age, quality, and graft size. Steatotic livers poorly tolerate IRI, leading to frequent and more severe HCC recurrence compared to transplants with non-steatotic grafts [83,84]. The impact of donor age on HCC recurrence after liver transplantation is still debated. Some studies have revealed a link between older donor age and recurrence [84], while others have not found a relationship [85]. Graft size is an important factor in living donor liver transplantation (LDLT). Studies have shown that small grafts with a low graft to recipient weight ratio of <0.7 or <0.8 promote HCC recurrence, especially in patients who are beyond established criteria [86,87]. The development of small-for-size syndrome, defined as graft dysfunction within the first week with patent vessels and no other etiology, may contribute further to tumor recurrence. First, LDLT grafts experience IRI during donor surgery due to repeat pringle maneuvers performed to minimize blood loss. And secondly, the LDLT grafts are required to regenerate after transplantation to meet the metabolic recipient demand. This pro-inflammatory environment, instigated by liver regeneration and IRI, is rich in inflammatory mediators favoring tumor growth [57,87].

Recipient factors play another key role in HCC recurrence. Higher body mass index (BMI) and older age have been linked to increased HCC recurrence and impaired oncological results [16,88,89]. Patients with HBV-related HCC were found to have a higher risk [90]. Pre-transplant bridging therapy including trans-arterial chemoembolization may be associated with increased recurrence risk through underlying mechanisms which remain to be better understood [3,91]. Pre-transplant selection criteria, such as the Milan criteria, Metroticket 2.0, and UCSF, utilize tumor characteristics to determine which recipients would benefit most from transplant. Patients who are within commonly used selection criteria experience lower recurrence rates [15]. High levels of immunosuppression may trigger HCC recurrence [18].

Larger tumor size and number, elevated alpha fetoprotein levels, are associated with more HCC recurrence [3,7,13,14]. Microvascular tumor invasion is another key indicator of tumor aggression and may indicate the presence of CTC, which migrate to the pro-inflammatory milieu in the transplanted graft and trigger regrowth and recurrence.

## 5. Mitigation of Tumor Recurrence Through Machine Perfusion

Machine perfusion (MP) is an exciting development within the field of transplantation. MP has improved graft utilization through viability assessment and bridging various logistical challenges. In addition, MP allows organ reconditioning and testing the future role of novel therapies during perfusion, including defatting agents, cell-based therapies, gene and immune modulation [92]. The two primary ex situ techniques are normothermic machine perfusion (NMP) and hypothermic oxygenated perfusion (HOPE). Randomized controlled trials (RCT) have demonstrated less early allograft dysfunction (EAD) for both techniques. The HOPE approach was also found to improve graft survival [29]. In the context of different graft risk profiles in such RCTs, both techniques contributed to a reduction in biliary and overall complications. Early evidence demonstrated a potential role of MP to decrease cancer recurrence through reduction in IRI-associated inflammation.

### 5.1. Hypothermic Oxygenated Perfusion

**Figure 2:**
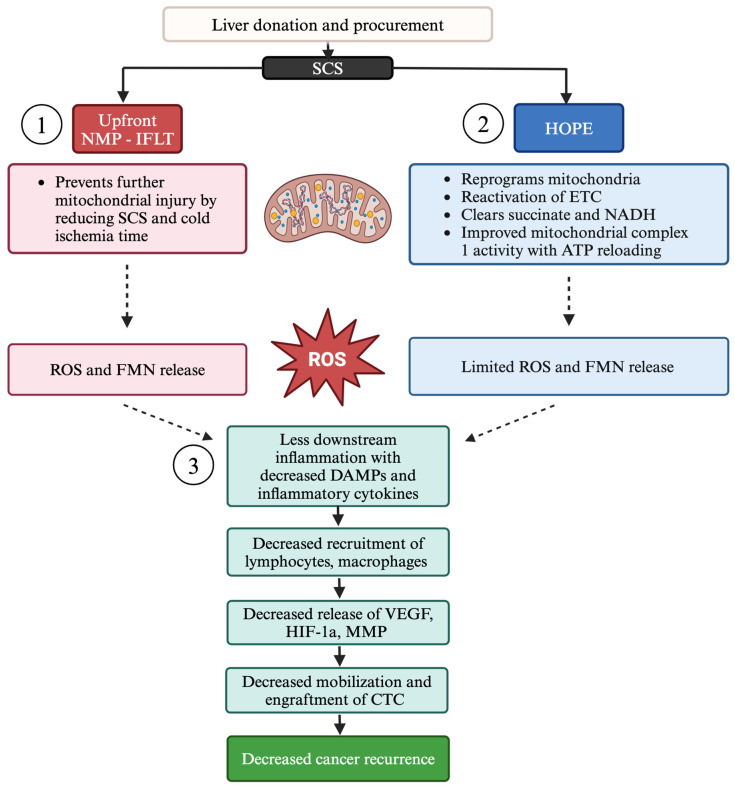
Decreased tumor recurrence with machine perfusion techniques. (1) Upfront normothermic machine perfusion (NMP) is utilized in “ischemia free liver transplantation” (IFLT). Immediate and continuous NMP without initial cold flush and cold storage maintain mitochondrial and other cell function to prevent further mitochondrial injury as seen with reperfusion under normothermic conditions after standard cold storage (SCS). As some injury already occurs in the donor, some reactive oxygen species (ROS) and flavin mononucleotide (FMN) are released from complex 1. The reduction in additional injury can reduce the downstream inflammation and cancer recurrence. (2) Hypothermic oxygenated perfusion (HOPE) reprograms the mitochondria and improves complex protein activity by restoring the electron transport chain (ETC) function. This allows for clearance of NADH and respiratory substrates such as succinate with concomitant ATP recharging. The direct consequence is a significant decrease in ROS and FMN release from complex 1 at implantation or subsequent NMP. (3) Downstream inflammation is also reduced with less DAMPs and cytokine release and less recruitment of pro-inflammatory cells. The releases of mediators like vascular endothelial growth factor (VEGF), hypoxia inducible factor (HIF-1a), and membrane metalloprotease (MMP) are reduced and there is decreased mobilization and engraftment of circulating tumor cells (CTC). This results in decreased tumor recurrence. Created with BioRender.com.

Hypothermic oxygenated perfusion (HOPE) delivers high oxygen concentrations to the liver at 8–12 °C. This modality can be performed through the portal vein alone (HOPE) or through both the portal vein and hepatic artery (D-HOPE). HOPE was found to reduce EAD, overall biliary complications, and re-transplantation rates compared to SCS. In addition, HOPE improves post-transplant graft survival in extended criteria donor livers [93,94,95]. By introducing oxygen under hypothermic conditions, HOPE can restore aerobic metabolism without release of high quantities of ROS. During ischemia, mitochondrial complex functions are on hold as the electron transport through the chain (ETC) stops. When oxygen is reintroduced, complex II tries to immediately reduce the high succinate levels causing an uncoordinated electron flow with subsequent ROS release at complex I. This mechanism is significantly reduced under cold conditions, thereby helping to reprogram mitochondria, metabolize succinate and NADH, and rebuild ATP. This remodeling prepares mitochondria for the subsequent rewarming and reperfusion under normothermic conditions at liver implantation. Together with ROS, flavin mononucleotide (FMN) dissociate from its pocket in complex I, enabling monitoring of complex I (and mitochondrial) dysfunction due to its autofluorescent abilities [96]. Accumulated TCA cycle byproducts become metabolized during HOPE, which significantly mitigates ROS release and IRI after transplant and prevents downstream inflammation and subsequent tumor recurrence (Figure 2).

Three main studies (Table 1) have examined the impact of HOPE on HCC recurrence until today. Mueller et al. performed a retrospective multicenter study that matched DCD grafts treated with HOPE with cold-stored DBD and DCD grafts. Recurrence rates of HCC were significantly lower in HOPE-treated patients, with 5-year recurrence-free survival (RFS) of 92% with HOPE compared to 81.2% with SCS [97]. Dajti et al. re-demonstrated decreased risk of HCC recurrence with HOPE compared to SCS [98]. In contrast, Rigo et al. showed a comparable RFS with HOPE and SCS but noted a higher utilization of extended criteria livers in the HOPE arm with comparable post-operative outcomes [22]. Such protective effects were seen despite higher donor risk (i.e., older donors, longer donor warm ischemia times) in most HOPE groups compared to cold storage controls (Table 1). The HOPE approach was also found to increase cell energy through better function of the respiratory chain. ATP recharging and less IRI were demonstrated to promote liver regeneration after transplantation of partial grafts [99,100]. Clinical studies are currently ongoing to examine the impact of HOPE on IRI in partial grafts compared to LDLT. A recent retrospective study examined post-transplant outcomes in pediatric recipients with HOPE-treated partial grafts, SCS partial grafts, and LDLT. Similar levels of IRI and post-reperfusion syndrome were observed with HOPE-treated grafts compared to LDLT grafts [101]. A multicenter RCT in France will further evaluate the impact of HOPE on IRI in partial grafts and in comparison to LDLT.

These studies have led to initiation of three European RCTs comparing the impact of end-ischemic HOPE and static cold storage alone on HCC recurrence and RFS (Modena/Italy: NCT06236568, Bologna/Italy: NCT05876052, in preparation: IICT multicenter and multinational trial initiated by Basel/Zurich in Switzerland) [19].

### 5.2. Normothermic Machine Perfusion Techniques

In contrast to HOPE, NMP oxygenates the graft with a blood-based perfusate at 37 °C. NMP also allows for viability assessment using perfusate and bile parameters. NMP can be initiated following a period of SCS (end-ischemic NMP) or upfront immediately following procurement after a short cold storage to prepare the graft and the device. “Ischemia free liver transplantation” (IFLT) is a technique that completely circumvents the need for cold flush and storage. Following donor liver mobilization, a segment of the donor external iliac vein is procured and used for a portal vein extension connecting the donor liver to the NMP device. The splenic artery and inferior vena cava are used to completely cannulate the liver, which is removed and implanted maintaining NMP [102].

Upfront and end-ischemic NMP and IFLT were found to reduce EAD compared to cold storage in RCTs [102,103,104,105]. Studies have shown that NMP stabilizes the mitochondrial membrane, decreasing release of cytochrome C, and induces a steady state of mitochondrial function [106]. In addition, NMP decreases pro-inflammatory gene expression and IRI when applied instead of cold ischemia storage (Figure 2) [107]. However, NMP is unable to reprogram mitochondria to tolerate high succinate loads and prevent FMN release, resulting in oxidative stress and damage [96]. Tang et al. performed a retrospective study to examine HCC recurrence in IFLT compared to a matched cold storage cohort. Three-year RFS was significantly improved with IFLT at 86.7% compared to 53.6% in the control group (Table 1) [33]. Currently, there are no further studies that have investigated HCC recurrence with upfront or end-ischemic NMP or normothermic regional perfusion.

## 6. Other Strategies to Reduce IRI and Mitigate Cancer Recurrence

### 6.1. Pharmacological Agents

#### 6.1.1. Anti-Inflammatory Agents

N-acetyl cysteine (NAC) is commonly used to reverse liver injury from acetaminophen toxicity by repletion of glutathione, a potent antioxidant. Several studies have examined the impact of NAC applied in the donor during procurement and in the recipient at transplant. A recent meta-analysis included thirteen studies with 1121 participants, 550 of whom received NAC in various modes, including the donor, recipient, or both. A protective effect against LT-induced IRI was observed, with decreased incidence of PNF, postoperative complications, peak aspartate aminotransferase (AST) level, and peak alanine aminotransferase (ALT) level. NAC also improved 2 y graft survival rates [108]. Another study showed that NAC administration during an-hepatic phase in recipients increased the expression of anti-inflammatory interleukin-4 (IL-4) and interleukin-10 (IL-10), protecting from IRI [109]. NAC and other molecules may therefore have a potential in also reducing HCC recurrence and might be tested in this context compared to machine perfusion techniques [110,111].

Prostaglandins are another important molecule class released by KC. The impact of Prostaglandin E1 (PGE1) on IRI has been investigated in multiple studies. PGE1 helps decrease platelet aggregation and release of inflammatory cells and induces vasodilation to improve hepatic blood flow [112,113]. The impact of PGE1 on HCC recurrence after liver transplant was examined in a retrospective trial by Kornberg et al. [114]. Alprostadil, a synthetic form of PGE-1, was given to patients in the intensive care unit until transfer to the regular floor or for a maximum of 7 days post-transplant. The study found that PGE-1 therapy decreased HCC recurrence, especially in patients exceeding the Milan criteria, thereby supporting the mechanisms described here.

#### 6.1.2. Adjuvant Systemic Therapy

Research on the impact of systemic chemotherapy after LT for HCC has not yielded promising outcomes. An RCT examined the effectiveness of post-operative adjuvant 5-fluorouracil, oxaliplatin, leucovorin regimen (FOLFOX) in HCC patients outside the Milan criteria [115]. Improved 1- and 3-year OS was observed (89.7% and 79.3% vs. 69% and 62.1%, respectively). However, no difference in recurrence rates at 1 and 3 years was found (44.8% and 51.7% with FOLFOX and 44.8% and 48.3% in control group) [115].

Sorafenib, a multikinase inhibitor, has been tested as an adjuvant treatment after LT for HCC. Early results showed promise with significant reduction in HCC recurrence [116], but other single-center case series did not confirm these findings [117]. The STORM trial examined the impact of adjuvant sorafenib on HCC recurrence after resection or ablation and found no significant difference in RFS [118].

Current evidence does not support adjuvant systemic therapies with chemotherapy or sorafenib to reduce the risk of HCC recurrence after LT [12]. Immune checkpoint inhibitors (ICI) have been a recent development in HCC treatment and the IMbrave 150 trial showed that atezolizumab (anti-programmable death ligand 1 (PDL1)) and bevacizumab (anti-VEGF) are an effective treatment for HCC [119]. Since then, Tremelimumab (anti-cytotoxic T lymphocyte-associated antigen 4 (CTLA-4)) and Durvalumab (anti-PDL1) have also been established as ICI therapies for HCC [120]. However, there are also concerns regarding the use of ICIs following transplant due to possible induction of graft rejection [121]. A recent systematic review examined ICI use in HCC patients. The study identified 15 patients who had ICI treatment for HCC recurrence after LT with 1 patient receiving ICI treatment prior to transplant. Of these 15 patients, 6 experienced fatal graft rejection and 12 patients died [122]. A recent multicenter retrospective cohort study sought to expand upon these findings [123]. Eleven centers were included with a total of 83 patients receiving ICI therapy prior to transplant, representing the largest cohort studied to date in this context. The ICIs utilized included Camrelizumab, Pembrolizumab, Sintilimab, Tislelizumab, Nivolumab, and Atezolizumab. Interestingly, Nivolumab and Atezolizumab, which are more commonly referenced in the literature, were among the least used in this study, with Nivolumab administered to only five patients and Atezolizumab to four. Acute rejection occurred in approximately 28% of the recipients, and six cases resulted in rejection-related deaths. A multivariate analysis identified a wash-out period of 30 days as the only significant protective factor against AR. These findings support considering a wash-out period with ICI use before proceeding with liver transplantation.

#### 6.1.3. Immunosuppression

Currently, there are no standardized protocols for the immunosuppression management of liver recipients with HCC. One of the most-used immunosuppression agents in liver transplant are calcineurin inhibitors (CNIs), such as tacrolimus and cyclosporine. Several studies have identified that CNI use is associated with a higher rate of post-transplant tumor recurrence, especially in a dose-dependent manner [18,124,125].

Mammalian target of rapamycin (mTOR) controls cell proliferation and survival, and HCC is associated with an upregulation of the mTOR pathway [126]. mTOR inhibitors (mTORi) such as sirolimus and everolimus have been increasingly used for immunosuppression in HCC patients. Several retrospective studies and meta-analyses indicate that sirolimus and everolimus reduce recurrence and enhance long-term survival compared to CNIs [127,128,129]. The SiLVER trial, a phase 3 RCT, examined the impact of treatment with mTORi on HCC recurrence post-transplant. RFS was improved 3 years after transplant with the highest benefit in younger patients and those within the Milan criteria [130]. The latest meta-analysis, which included 23 comparative studies (17 observational and 6 randomized controlled trials) involving 6495 patients, found that mTORi-based therapy significantly improved RFS and OS at 1 and 3 years, with no significant increase in acute rejection episodes [131].

While there is no standard post-transplant immunosuppression protocol for HCC, a current consensus recommends using steroid-free immunosuppression and decreasing CNI exposure by incorporating mTOR inhibitors due to their anti-tumor potential [132].

#### 6.1.4. Immunotherapy

Studies have examined the role of immunotherapy in preventing HCC recurrence following transplant. The innate and adaptive immune system play a key role in immunosurveillance and prevention of cancer. In transplant, the adaptive immune system is suppressed to prevent rejection of the new graft. However, the innate immune system remains active and methods to potentiate the immunosurveillance role of the natural killer (NK) cell have been studied. Ohira et al. describe isolation of donor liver-derived NK cells to prevent HCC [133,134]. Eighteen patients underwent transplant for HCC and received donor liver NK cell infusion at 3–5 days after transplant. The cohort was divided into two groups based on the number of cells received during the infusion. The study found no HCC recurrence within their cohort and improved OS in the high-dose group, with a follow-up period of more than 24 months. No signs of acute rejection or graft versus host disease were noted. Further studies are needed to assess the impact of immunotherapy on HCC recurrence.

### 6.2. Other Surgical Interventions

Two different approaches to precondition liver tissue intraoperatively to reduce IRI have emerged: ischemic preconditioning (IPC) and remote ischemic preconditioning (RIPC). IPC is performed via the Pringle maneuver occluding the liver inflow for a short period followed by repeat reperfusion. RIPC is a simpler procedure and consists of an intermittent pneumatic peripheral compression of a limb. A recent meta-analysis compared both procedures, including a total of nine RCTs (seven IPC and two RIPC studies) [135]. The study revealed improvement in postoperative AST with a statistically lower mortality rate in the IPC group (*p* = 0.04). In the case of RIPC, the data are currently insufficient. An RCT including 208 participants analyzed the effect of RIPC on both the donors and recipients following pediatric LT. RIPC did not significantly improve ALT and AST levels among donors and recipients or impact on the incidences of EAD, PNF, and post-transplant complications [136]. Additionally, an animal study performing IPC in mice has demonstrated a decrease in overall tumor burden in ischemic steatotic livers to levels comparable to those in healthy controls [137]. No studies have examined the clinical impact of IPC on cancer recurrence.

## 7. Summary and Future Directions

Ischemia-reperfusion injury creates a pro-inflammatory milieu that supports tumor cell resettling and engraftment, playing an important role in HCC recurrence after liver transplantation. Minimizing reperfusion injury before organ implantation seems to be an effective strategy for decreasing tumor recurrence. In this review, we have outlined how machine perfusion may decrease IRI and HCC recurrence. HOPE allows for mitochondrial reprogramming to decrease IRI and has been shown to improve RFS following transplant for HCC patients. IFLT provides continuous NMP and has also been shown to reduce HCC recurrence. Several pharmacological and surgical strategies may supplement the benefit provided by machine perfusion.

The management of primary and secondary liver cancers is expected to evolve significantly, not only with better downstaging and retransplant disease control, but also with the wider adoption of machine perfusion technologies, enabling the transplantation of more candidates with different liver tumors. Machine perfusion technologies allow organ assessment, increasing the safe utilization of marginal grafts. More clinical studies are however needed to verify the long-term benefits of machine perfusion on cancer recurrence and post-transplant complications. Future studies should focus on underlying mechanisms of protection though MP, the standardization of perfusion protocols, and the impact on recurrence rates and long-term survival. The safe use of riskier organs through viability assessment will unlock the currently poorly utilized donor pool, enabling transplantation of more candidates with various liver cancer types.

## Figures and Tables

**Table 1:** Overview on clinical studies assessing the impact of machine perfusion on cancer recurrence after liver transplantation.

Study	Study Design and Country	Perfusion Techniques (Device) and Duration	Donor Type	Donor Risk Factors	Outside HCC Transplant Criteria	Follow-Up	HCC Recurrence Rate	Key Findings
Clinical studies with hypothermic oxygenated perfusion								
Mueller et al., 2020 [97]	Retrospective multicenter - Center A: Switzerland - Center B: United Kingdom	HOPE (XVIVO Liver Assist) (*n* = 70) with 2 h (IQR:1.7–2.5) SCS (*n* = 210)	DCD treated with HOPE: *n* = 70 (Center A) DBD control: *n* = 70 (Center A) DCD control: *n* = 70 (Center B) DBD control: *n* = 70 (Center B)	Donor age was significantly higher in HOPE group (*p* = 0.007) 59.5 years (IQR: 48.75–72.0) FDWIT was significantly higher in HOPE group (*p* < 0.0001) 30.5 min (IQR: 26–35)	Milan: HOPE: 25/70 (35.7%) DCD control: 13/70 (18.6%) DBD control (A): 26/70 (37.1%) DBD control (B): 14/70 (20%) UCSF: HOPE: 20/70 (28.6%) DCD control: 6/70 (8.6%) DBD control (A): 21/70 (30%) DBD control (B): 4/70 (5.7%) Metroticket 2.0: HOPE: 13/70 (18.6%) DCD control: 1/70 (1.4%) DBD control (A): 12/70 (17.1%) DBD control (B): 1/70 (1.4%)	HOPE: 32 mo (15.8–55.6) DCD control: 54 mo (31.5–68.8) DBD control (A): 54.3 mo (24.6–87.8) DBD control (B): 44.9 mo (27.4–67.5)	DCD-HOPE: 4/70 (5.7%) DCD control: 10/70 (14.3%) DBD control (Center A): 18/70 (25.7%) DBD control (Center B): 12/70 (17.1%)	Fourfold higher tumor recurrence in unperfused DBD livers (25.7%, 18/70), compared to only 5.7% recipients (4/70) with tumor recurrence in the HOPE-treated DCD cohort (*p* = 0.002) in Center A and twofold higher tumor recurrence rate in unperfused DBD and DCD from Center B. Five-year RFS: 92% in HOPE group, compared to 73%, 82.7%, and 81.2% in patients receiving unperfused DBD or DCD livers.
Rigo et al., 2023 [22]	Retrospective single center; Italy	D-HOPE (XVIVO Liver Assist) (*n* = 80) with 2.4 h (1.95–3) SCS (*n* = 246)	D-HOPE: DCD—14/80 (18%); DBD—66/80 (82%) SCS: DCD: 0%; DBD: 246/246 (100%)	Donor age was significantly higher in HOPE group 71.8 years (IQR: 60.7–82.4, *p* = 0.003), FDWIT not reported	Metroticket 2.0, <80% estimated survival at 5 years D-HOPE: 8/80 (10%) SCS: 20/246 (8%) Downstaging proportion comparable (*p* = 0.673)	D-HOPE: 40 mo (32–52) SCS: 59 mo (36–72)	D-HOPE: 8/80 (10%) SCS: 22/246 (9%)	Similar 1-year RFS: 96% SCS, 95% D-HOPE. Adjusted analysis for donor age, donor BMI, macrovesicular steatosis, CIT, recipient age, HCC grading, and MVI showed no significant difference in RFS.
Dajti et al., 2024 [98]	Retrospective single center; Italy	HOPE (instituitional device) (*n* = 60) SCS (*n* = 177)	HOPE: DCD—16/60 (27%); DBD—44/60 (73%) SCS: DCD: 0%; DBD: 177/177 (100%)	Donor age was comparable (*p* = 0.2) FDWIT not reported	Milan: HOPE: 1/60 (7%), 17/60 (28.3%) outside Milan, 10/60 downstaged SCS: 22/177 (10%), 51/177 (28.8%) outside Milan, 29/177 downstaged	HOPE: 32 mo (24–48) SCS: 45 mo (27–64)	At 2 years: HOPE: 1/60 (2%) SCS: 14/177 (8%)	HOPE associated with lower risk of HCC recurrence (OR 0.126, *p* = 0.049) and higher RFS (HR 0.132, *p* = 0.050) after balancing recipient age, sex, MELD, DRI, and Milan criteria status at listing. MVI (OR 3.737, *p* = 0.019) and longer CIT (OR 1.155, *p* = 0.049) were associated with higher risk of recurrence.
Clinical studies with ischemia-free liver transplantation								
Tang et al., 2021 [33]	Retrospective single center; China	IFLT (XVIVO Liver Assist) (*n* = 30) CLT (*n* = 85)	All donors were DBD	Donor age was comparable (*p* = 0.117)	Milan: IFLT: 13/30 (43.3%) CLT: 44/30 (51.8%) AFP is lower in IFLT group (*p* = 0.016)	IFLT: 22.9 ± 11.2 mo CLT: 22.6 ± 11.4 mo	At 1 year, RFS IFLT: 92.2% SCS: 88.1%	RFS at 1 and 3 year was significantly improved with IFLT: 92.2% and 86.7% vs. 88.1% and 53.6% for CLT (*p* = 0.048). Difference between OS at 1 and 3 years not statistically significant: IFLT 96.7% and 90.6%, vs. CLT 94.1% and 70.6%

BMI: body mass index; CLT: Conventional liver transplantation; CIT: cold ischemia time; DBD: donation after brain death; DCD: donation after circulatory death; HCC: hepatocellular carcinoma; HOPE: hypothermic oxygenated perfusion; IFLT: “ischemia free” liver transplantation; MVI: microvascular invasion; OS: overall survival; RFS: recurrence-free survival; SCS: standard cold storage.
